# An integrated approach of comparative genomics and heritability analysis of pig and human on obesity trait: evidence for candidate genes on human chromosome 2

**DOI:** 10.1186/1471-2164-13-711

**Published:** 2012-12-19

**Authors:** Jaemin Kim, Taeheon Lee, Tae-Hun Kim, Kyung-Tai Lee, Heebal Kim

**Affiliations:** 1 Interdisciplinary Program in Bioinformatics, Seoul National University, Seoul, Republic of Korea; 2 Department of Agricultural Biotechnology and Research Institute for Agriculture and Life Sciences, Seoul National University, Seoul, Republic of Korea; 3 Division of Animal Genomics and Bioinformatics, National Institute of Animal Science, Rural Development Administration, Suwon, 441-706, Republic of Korea; 4 C&K Genomics, Seoul National University Research Park, Seoul, 151-919, Republic of Korea

**Keywords:** Obesity, Synteny, Comparative genomics, Heritability, Back-fat thickness, Subscapular skinfold thickness, Chromosome 2, Pig, Human

## Abstract

**Background:**

Traditional candidate gene approach has been widely used for the study of complex diseases including obesity. However, this approach is largely limited by its dependence on existing knowledge of presumed biology of the phenotype under investigation. Our combined strategy of comparative genomics and chromosomal heritability estimate analysis of obesity traits, subscapular skinfold thickness and back-fat thickness in Korean cohorts and pig (Sus scrofa), may overcome the limitations of candidate gene analysis and allow us to better understand genetic predisposition to human obesity.

**Results:**

We found common genes including *FTO*, the fat mass and obesity associated gene, identified from significant SNPs by association studies of each trait. These common genes were related to blood pressure and arterial stiffness (P = 1.65E-05) and type 2 diabetes (P = 0.00578). Through the estimation of variance of genetic component (heritability) for each chromosome by SNPs, we observed a significant positive correlation (r = 0.479) between genetic contributions of human and pig to obesity traits. Furthermore, we noted that human chromosome 2 (syntenic to pig chromosomes 3 and 15) was most important in explaining the phenotypic variance for obesity.

**Conclusions:**

Obesity genetics still awaits further discovery. Navigating syntenic regions suggests obesity candidate genes on chromosome 2 that are previously known to be associated with obesity-related diseases: *MRPL33, PARD3B, ERBB4, STK39*, and *ZNF385B*.

## Background

Candidate gene approach has been proven to be an extremely powerful and effective method for studying the genetic architecture of complex traits. This approach is, however, criticized for non-replication of results when followed up in subsequent association studies. Traditional candidate gene approach is also greatly limited by its reliance on the priori knowledge about the physiological, biochemical or functional aspects of possible candidates, and unfortunately, the detailed molecular anatomy of most complex traits still remains veiled. Such limitation results in a fatal information bottleneck, and comparative genomics serves as an extended strategy to solve the problem of information bottleneck. This strategy makes the utility of cross-species approach to characterize the effect of putative candidate genes [1,2].

To date, several comparative analysis studies of human and pig on obesity-related traits have confirmed the human obesity genes affecting fatness traits of pigs (Additional file 1: Table S1). Pig is an exceptional biomedical model related to energy metabolism and obesity in humans [3]. It shares many physiological similarities with humans, making it an optimal species for preclinical experimentation as well [4]. Especially, the back-fat thickness (BFT) of pig is closely related to total body fat so that the study of this trait can provide a unique approach to understanding the causes of human obesity [5]. Fontanesi et al. found association of an SNP in *FTO* with intermuscular fat deposition in Italian Duroc pigs, confirmed in a later study by themselves and by a recent study of Fan et al. [6-8]. On the other hand, Du and collaborators found association between *TCF7L2*, one of type 2 diabetes genes, and BFT [9]. Nowacka-Woszuk et al. mapped 13 candidate genes in the pig genome and found most of them were located within known quantitative trait loci (QTL) to confirm the association with pig fatness traits [10].

Nevertheless, published comparative studies still do not break the barrier of information bottleneck, because it starts with presumed biology of one species and applies it to another species. Thus, we previously conducted local genomic *de novo* sequencing of a porcine QTL region affecting fatness traits to carry out SNP association study for BFT and related the result to human association study of subscapular skinfold thickness (SUB). This study allowed us to expand the QTL results observed in pig to human common forms of obesity, but it still is a hypothesis-driven genetic approach as it only considered known QTL region instead of the genome. In order to overcome the lack of thoroughness and inclusiveness that candidate gene approach is criticized for, we conducted the genome-wide comparative studies on common form of obesity traits.

Our study is also an integrated analysis, as it adopts the concept of the population-based heritability estimates which can provide a valuable metric of available genetic risk information [11]. GCTA (Genome-wide Complex Trait Analysis) tool implements the method of estimating variance explained by all SNPs and extends the method to partition the genetic variance onto each of the chromosomes. By fitting the effects of all the SNPs as random effects in a mixed linear model (MLM), this tool partially unveil the “missing heritability” problem caused by the inability to detect a large number of common variants with small effects and rare variants with large effects [12,13].

We integrated heritability analysis and comparative genomics strategy to both identify causal genetic factors in the pig genome and to expand the knowledge of genetic risk factors predisposing to common forms of obesity in humans. Combined strategy would provide a more powerful comprehensive means to counter the criticism of candidate gene studies.

The prevalence of obesity has increased greatly; it has tripled in the last five decades in America, and over 400 million people are obese [14,15]. The amount of fat in the body, adiposity, is regulated as the process of energy homeostasis, controlled by circulating signals related to the size of the fat mass (adiposity signals) integrated with signals from the gastrointestinal system (satiety signals). Adiposity signals are connected through central autonomic pathways to centers that process satiety signals such as cholecystokinin (CCK). These integrated signals are known to regulate the meal size and body fat [15]. Obesity can cause various healthcare problems, including type 2 diabetes, cardiovascular diseases, hypertension, etc. [3] For instance, it can lead to the development of insulin resistance, one of the reasons for pancreatic islet β-cell dysfunction and apoptosis, resulting in progression to impaired glucose tolerance, followed by the increased risk of type 2 diabetes [9,16].

Due to the considerable evidence that obesity, the worldwide epidemic, is highly heritable [17], numerous studies including Genome-wide association studies (GWAS) have elucidated much of genetic architecture of obesity. Despite extensive efforts to the search of obesity at gene and nucleotide levels, considering the substantial heritability estimate of 40 to 70% [18], further research is still needed. To this aim, we analyzed obesity related traits of human and pig on genome-wide scale in cross-species approach to identify potential genes susceptible to human obesity disease. We present results of our combined approach of comparative and chromosomal heritability estimate analysis in an effort to elucidate the genetic basis of human obesity.

## Methods

### Sequence data

We used the US National Center for Biotechnology Information (NCBI) site as the source of the H. sapiens genomic sequence (version GRCh37.p5) and Sscrofa10.2 for Pig (Sus scrofa) genome.

### Alignment strategy

The pig genome was mapped onto the human genome, using a large-scale alignment tool, LAST [19]. The chromosomal summary of autosomal pig SNPs mapped onto Sscrofa10.2 is described in (Additional file 2: Table S5). We used the default DNA scoring scheme: match score of 1, mismatch cost of 1, cost of 7 for gap, and gap extension cost of 1. The minimum alignment score was set at 150. Using the sorted alignments (“maf-sort.sh” script), we finally proceeded to “maf-cull” step to remove redundant alignments. The synteny map was drawn with Circos software [20] using Bundling Links function. We only considered autosomal chromosomes for this study, and the number of syntenic SNPs of pig and human by each chromosome is described in (Additional file 2: Table S4).

### Genotype

The genomic DNAs of pig were genotyped on the Illumina Porcine 60 K SNP Beadchip. We discarded the markers with low MAF (<0.01), significant deviation from Hardy-Weinberg equilibrium (P < 10^-3^), and low genotype call rate (<95%). This quality-control process left 45,013 autosomal SNPs. The SNP probes were mapped on Sus Scrofa genome 10.2 from NCBI FTP using BLAT [21].

Using the synteny information provided by LAST alignment tool, we filled each human chromosome regions with corresponding DNA segments of pig and their defined SNPs. Therefore, we defined the pig genome in human chromosome level.

The genomic DNAs of human were genotyped on the Affymetrix Genome-Wide Human SNP array 5.0 containing 500,568 SNPs. Markers (GRCh37) with high missing gene call rate (>5%), low MAF (<0.01) and significant deviation from Hardy-Weinberg equilibrium (P < 10–6) were excluded, leaving a total of 326,262 markers to be examined in 8,842 individuals.

### Phenotype

We analyzed inbred Berkshire population, and a total of 14 meat quality traits were measured. Traits include back-fat thickness, carcass weight, meat pH, meat color, muscle shear force, drip loss, heat loss, water holding capacity, and intramuscular fat content, etc. The back-fat thickness, which we used for this specific study, was measured between the 10th and 11th rib. The phenotype (back-fat thickness) was adjusted by the age effect using the linear model of y = b_0_ + b_1_ × age + e, and then we standardized the residuals to z-scores, in each sex group separately. We used the age data (days) at the time of slaughter. After excluding samples that do not have age information, we examined in 697 samples.

The subscapular skinfold thickness (human) was also adjusted by age and standardized in each gender and area group (rural and urban cities). Subscapular skinfold thickness is a measurement for upper body fat distribution. It is measured just below the angle of the left scapula with the fold either in a vertical line or slightly inclined [22]. The sampling base for both cohorts is in Gyeonggi Province, close to the capital of the Republic of Korea. We used the data of Korean cohorts (KARE) of 8,842 individuals aged 40 to 69 and analyzed 8,801 samples that had SUB phenotype data available.

### GCTA & GWAS

We used the GCTA tool [12] to calculate heritability for SUB and BFT. We calculated the genetic relationship matrix (GRM) between all pairs of samples using all the autosomal SNPs by “make-grm” option. We then estimated the variance of genetic component, or heritability, for each trait by restricted maximum likelihood analysis. We also estimated variance explained by each chromosome using joint analysis (by multiple GRMs option).

Linear regression analysis was performed in an additive model with the data adjusted by sex and age using PLINK-linear option [23]. GWAS have important limitations, such as the potential for false-positive and false-negative results and for biases related to selection of study participants and genotyping errors [24]. Hence, we may need to be careful to interpret single GWA result. For these factors taken into consideration, we investigated markers significantly identified commonly from both human and pig, although pig data had its own limitations of relatively small population size and low number of SNPs. The Database for Annotation, Visualization and Integrated Discovery (DAVID) v6.7 was used to determine gene-disease association [25,26].

## Results and discussion

**Figure 1:**
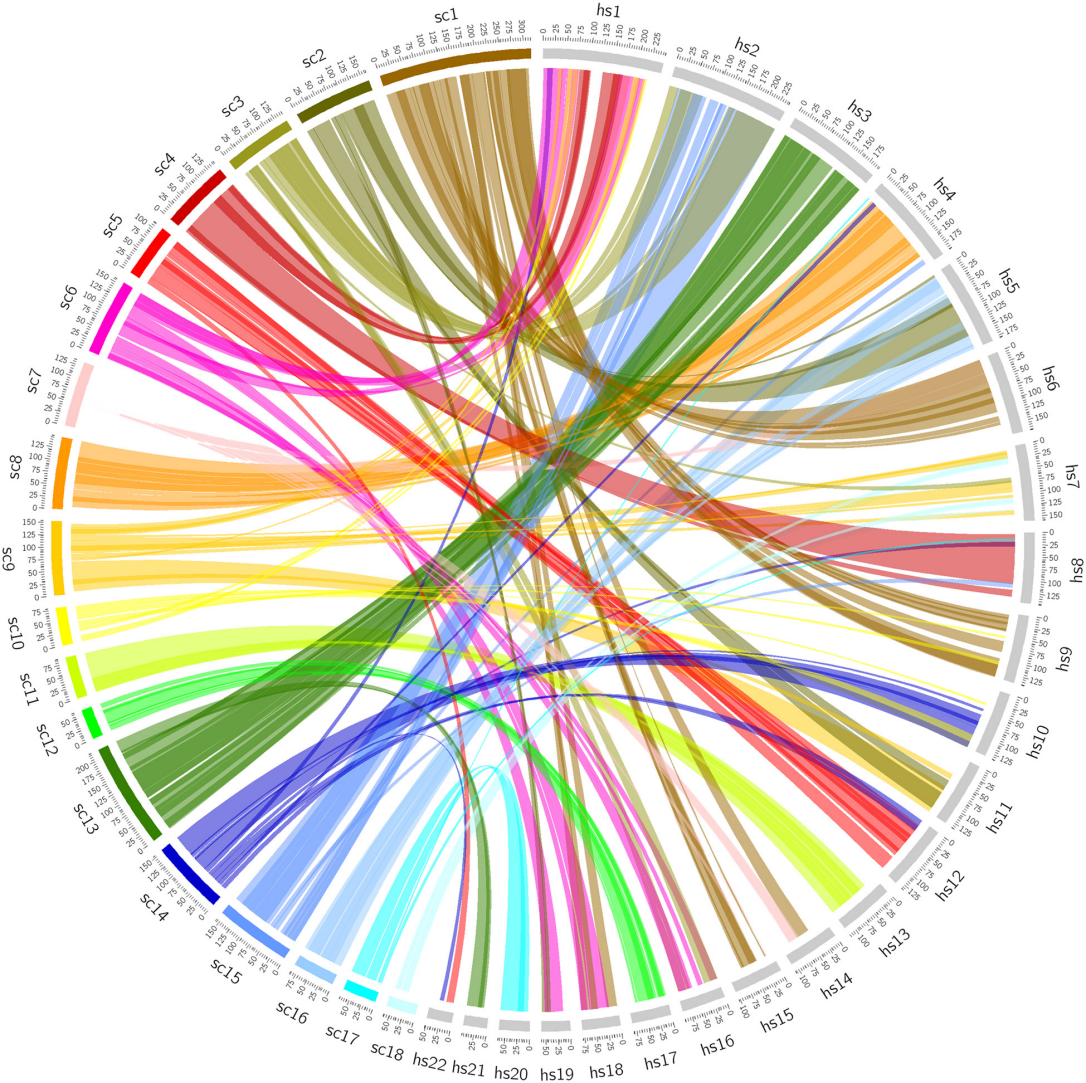
**A synteny map of the pig and human genomes.** The synteny map represents 18 pig (sc) and 22 human (hs) autosomal chromosomes. It was drawn using Circos software. Human chromosome 2, for example, is mostly syntenic to pig chromosomes 3 and 15.

We mapped the pig genome onto the human genome using the alignment tool (LAST) and found the syntenic region; the pig chromosomes are rearranged in human chromosome scale. The synteny map represents 22 and 18 autosomal chromosomes of human and pig (Figure 1 and Additional file 2: Table S3). Syntenic regions of each chromosome of each species are linked with same bundle color. This result is corroborated by previous work of cytogenetic map [27]. For our analysis later, we mention briefly here that human chromosome 2 is syntenic to pig chromosomes 3 and 15 mostly.

**Figure 2:**
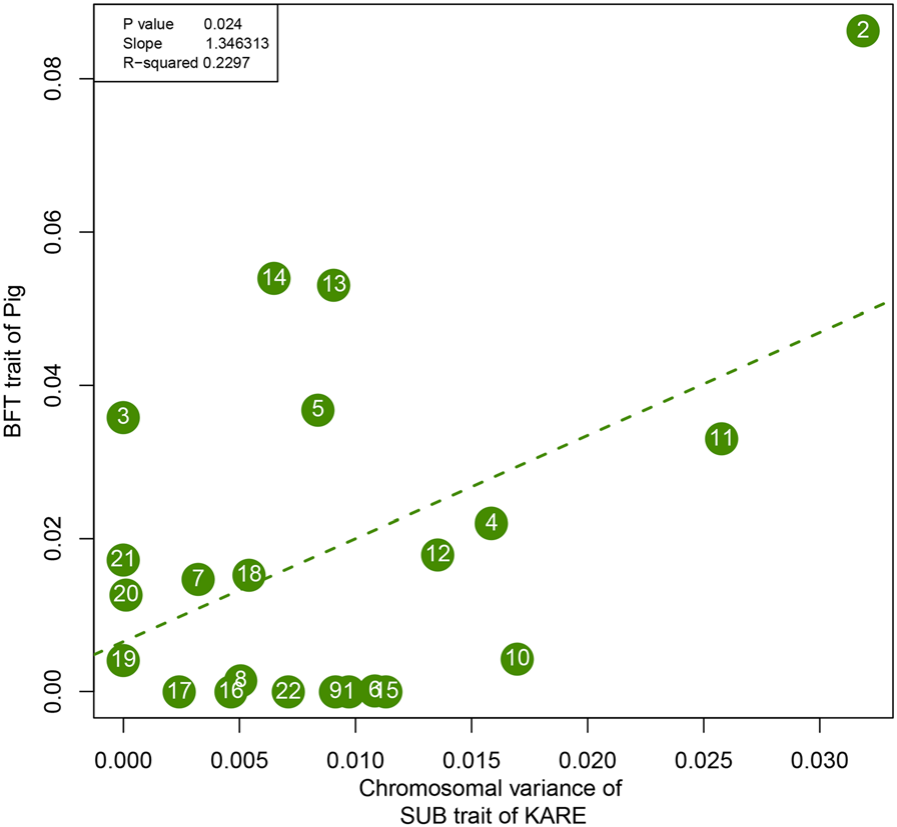
**Chromosomal heritability analysis results of pig versus human.** Shown is the estimate of the variance explained by each chromosome for SUB against that for BFT. The regression slope and R-squared are 1.9088 and 0.2104. Although correlation is not perfect (r = 0.479), a significant positive slope (P = 0.024) indicates that chromosomes that explain higher variance for BFT in pig tends to contribute more to SUB in human.

We then estimated the heritability of BFT of pig and SUB of human on each chromosome by SNPs, using GCTA tool (Additional file 3: Table S2) [12]. Out of 45,053 autosomal SNPs for 697 pig samples, we used 16,123 SNPs and 78,926 SNPs from a total of 326,262 SNPs for 8,801 Korean cohorts. We observed a significant positive correlation between chromosomal variance of BFT of pig and SUB of human (Figure 2). Although the correlation was not perfect (correlation coefficient of 0.479), according to Williams [28], a correlation coefficient between 0.4 and 0.7 is considered as a substantial relationship. We also found a significant positive slope (slope = 1.346, P = 0.024). These sufficiently indicated that the chromosomes that explain higher variance on pig trait tend to have higher heritability estimates for human trait as well. Especially, chromosome 2 (syntenic to pig chromosomes 3 and 15) seemed to be the most important chromosome in affecting the obesity risk from both populations, leaving detailed analysis on chromosome 2.

Considering the significantly correlated genetic contribution to obesity between human and pig, we performed GWAS and extracted 795 common genes detected from significant SNPs achieving a nominal p-value < 0.05 (Table 1). We found that common genes evidently caused obesity-related diseases such as blood pressure and type 2 diabetes (P = 1.65E-05, 0.00578, respectively) enriched by DAVID [25,26]. The genes included the fat mass and obesity associated gene *FTO* with corresponding SNPs of rs13337356 (P = 0.0157) on human chromosome 16; H3GA0017791 and INRA0021406 (P = 0.01022 and 0.007, respectively) for pig on chromosome 6. This gene encodes for 2-oxoglutarate-dependent nucleic acid demethylase of which expression in the brain seems regulated by feeding and fasting, and it is associated with increased lipolytic activity in adipocytes [6]. Another major obesity gene *GNPDA2* was associated with both traits (Additional file 4: Table S6). These demonstrate that conserved genetic regions of two species maintain the function to alter the level of obesity. As we look closely to each population, especially for Berkshire, we identified additional variants in the genes associated with obesity, evidenced from multiple studies: *RASAL2, NPC1, INSIG2* (Additional file 4: Table S6 and Additional file 5: Figure S1).

**Table 1:** Common genes - disease association enriched by DAVID tool

**Disease Term**	**Genes**	**P-value**
Framingham Heart Study 100 K Project: genome-wide associations for blood pressure and arterial stiffness	*SLC9A9, CDH13, CAMK4, TNR, GPC6, EXOC4, C14ORF118*	1.65E-05
Meta-analysis of genome-wide association data and large-scale replication identifies additional susceptibility loci for type 2 diabetes	*FTO, JAZF1, TSPAN8, CDKAL1, TCF7L2, CAMK1D*	0.00578

**Figure 3:**
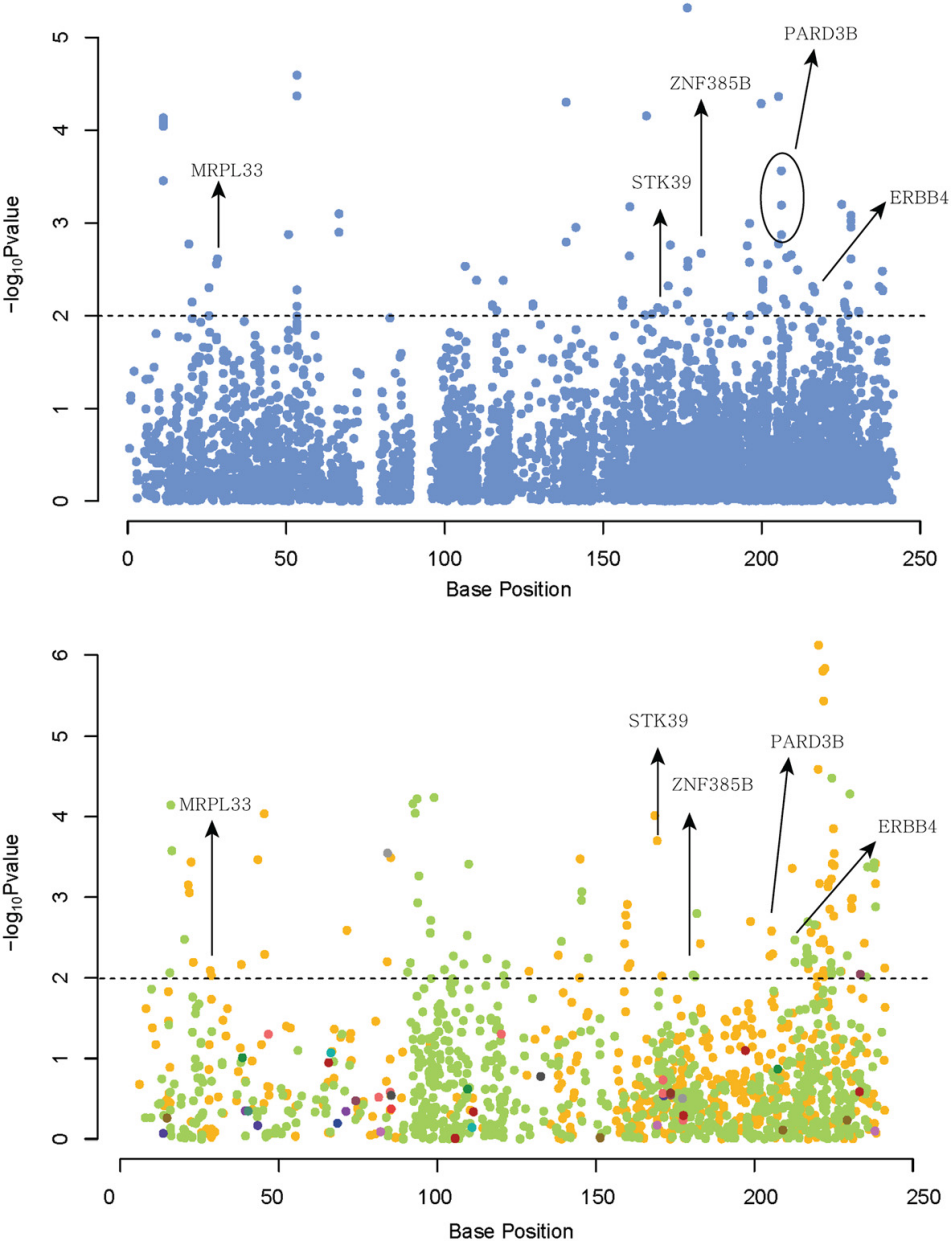
**Genome-wide association study results of pig and human on chromosome 2.** GWAS results are plotted as negative log-transformed P values associated for SUB (above) and BFT. Common genes were identified with SNPs that exceeded the significance threshold of 0.01. The orange points denote the region syntenic to pig chromosome 3; green ones to pig chromosome 15. Other colours represent minor syntenic portions other than chromosome 3 and 15.

The numerous genes found to be associated to obesity or obesity-related diseases from previous studies still contribute only a small fraction of the variance, meaning that obesity genetics still awaits further discovery. From Figure 1, we noted that chromosome 2 was the most important chromosome in explaining the variance of this trait. Therefore, we hypothesize that chromosome 2 plays a major role to the risk of obesity. The previous studies have found several obesity genes on chromosome 2 including *TMEM18, POMC, GCKR*, and *INSIG2*[14,29,30]. Our study could not detect these genes via GWAS as some of which are not in syntenic regions to be analyzed. However, we observed the similar peak patterns of graphical summary of genome-wide association results between two species (Figure 3). Although the plot was not dense enough, especially for back-fat thickness of pig due to relatively small number of syntenic markers, there were some regions that showed some similarities. This implies that human chromosome 2 and pig chromosomes 3 and 15 have conserved regions highly responsible for the level of obesity. We found the common nearest genes from variants reaching the significant threshold of P < 0.01 (Table 2). All the variants were located in intron region. Here we applied a more stringent threshold to present obesity genes with higher statistical significance. We suggest obesity candidate genes such as *MRPL33, PARD3B,* and *ERBB4* with evidence of association with type 2 diabetes. Also, hypertension and sudden cardiac arrest revealed to be affected by common significant genes on chromosome 2: *STK39 and ZNF385B*. Further evaluation of these results may help researchers to elucidate the genetic predisposition to obesity, a major threat to the public health.

**Table 2:** Genes identified on chromosome 2 that possibly alter obesity risk

**Genes**	**variants**^**a**^	**Position**^**a**^	**p-value**	**Function**	**Related Diseases**	**Reference**
*MRPL33*	DBWU0000918	118300227(3)	0.008089	Mammalian mitochondrial ribosomal proteins	Type 2 Diabetes Mellitus	[29,31]
	rs4666014	28019175	0.002452			
*STK39*	MARC0089023	72257469(3)	0.000199	Serine/threonine kinase	Hyper-tension	[32-34]
	rs10497337	169027149	0.008838			
*ZNF385B*	ALGA0088337	94656675(15)	0.009191	zinc finger protein	Sudden cardiac arrest	[35]
	rs17824619	180608843	0.002138			
*PARD3B*	MARC0060764	109267583(3)	0.005037	cell cycle/cell division	Type 2 Diabetes, chronic kidney disease	[36]
	rs7568370	205899090	0.000275			
	rs1510772	205934654	0.001348			
	rs4675490	205942560	0.000644			
*ERBB4*	DRGA0015447	126154685(15)	0.003372	Tyr protein kinase	Diabetes	[37]
	ALGA0086849	126191456(15)	0.006382			
	rs16848144	213141299	0.008011			

^a^ rs denotes human markers ^b^ syntenic pig chromosome in parentheses.

Measurement error in assessing skinfold thickness in human may be considerably large, and this likely resulted in lower statistical power than that for pigs [3]. Because we only analyzed the sytenic regions of the genome of each species with limited sample size, we had the increased sampling error for the heritability estimate and the reduced power of a conventional GWAS. Also, the size of linkage disequilibrium for pig is known to be much larger than that for human [38], and this can be a confounding effect when detecting causal variants for pig. Finally, the sequence difference among species especially on nonconding regions is huge that prospective SNPs might not have been identified [8].

Racial differences in genetic effects for complex traits are frequently debated in clinical and molecular research [39], and thus our research may have resulted differently if European cohorts were considered instead of Koreans. As much of the work in GWAS has focused on European populations, extending GWAS to different populations may provide new discoveries. In addition, various fat-related traits, including BMI of human and intramuscular fat content of pig, can be evaluated for this type of comparative studies; however, in order to make direct comparison between two species of common traits possible, we focused our research specifically on subscapular skinfold thickness and back-fat thickness.

## Conclusions

This work demonstrates a new approach of comparative study as it adopts the concept of an important parameter in genetics, heritability. Heritability is the proportion of phenotypic variation that is attributed to genetic components and thus provides insights into the biological significance of a certain trait. We observed that human chromosome 2 (SSC 3 and 15) explained the largest proportion of heritability for common obesity traits. Therefore, we hypothesized that chromosome 2 is crucial for remained complexities of genetic architecture of obesity. Based on this knowledge, we further investigated this chromosome to suggest candidate markers and genes that possibly control obesity.

## Abbreviations

SUB: Subscapular skinfold thickness; BFT: Back-fat thickness.

## Competing interests

The authors declare that they have no competing interests.

## Authors’ contributions

JK designed, analysed the data and wrote manuscript. TL performed the analysis. TK and KL collected the samples and genetic data. HK conceived and designed the analysis. All authors read, commented and approved the manuscript.

## Supplementary Material

Additional file 1 **Table S1.** Comparative analysis studies of human and pig on obesity-related traits. Click here for file

Additional file 2 **Table S3.** A Synteny table of the pig and human genomes. **Table S4.** Number of syntenic SNPs of pig and human by each chromosome. **Table S5.** Number of autosomal pig SNPs mapped onto Sscrofa 10.2 by each chromosome. Click here for file

Additional file 3 **Table S2.** Summary of heritability estimates by each chromosome for SUB and BFT traits. Click here for file

Additional file 4 **Table S6.** Major genes predisposing to common obesity identified from multiple previous works and our study. Click here for file

Additional file 5 **Figure S1.** Graphical summary (Manhattan plot) of genome-wide association results of SUB (above) and BFT. *RASAL2*, known as obesity gene, was significantly associated in SUB trait of pig. Click here for file
