# Supplementary material for: An integrated approach of comparative genomics and heritability analysis of pig and human on obesity trait: evidence for candidate genes on human chromosome 2
Source: BMC Genomics. 2012 Dec 19;13:711. doi: 10.1186/1471-2164-13-711 (PMC3562524; doi:10.1186/1471-2164-13-711)
Supplement: Additional file 1 — Table S1. Comparative analysis studies of human and pig on obesity-related traits. [file 1471-2164-13-711-S1.pdf]

Table S1 - Comparative analysis studies of human and pig on obesity-related traits

Gene	Method	Trait [a]	Breeds	Reference
FTO	SNP association	VIF, BFT, LC	Italian Duroc and commercial pigs	Fontanesi [7]
FTO	SNP association	VIF	Italian Duroc	Fontanesi [6]
FTO	SNP association	IFC and growth rate	Berkshire × Yorshire	Fan [8]
FTO	Expression studies (qPCR)	–	Gottingen minipig,	Madsen [33]
TCF7L2	SNP association	BFT, TOTLIPPR	Berkshire × Yorshire	Du [9]
INSIG2, LIPIN1, NR3C1, etc.	Chromosomal localization (FISH) and QTL	BFT and lipid content	Porcine BAC library	Nowacka-Woszuk [10]
NEGR1, FAM73A, TTLL, etc.	SNP association	Subcutaneous fat thickness	Korean native × Landrace	Lee [3]

[a] BFT, back fat thickness; VIF, visible intermuscular fat; LC, lean cuts; IFC, intramuscular fat content; TOTLIPPR, total lipid percentage
