# Supplementary material for: An integrated approach of comparative genomics and heritability analysis of pig and human on obesity trait: evidence for candidate genes on human chromosome 2
Source: BMC Genomics. 2012 Dec 19;13:711. doi: 10.1186/1471-2164-13-711 (PMC3562524; doi:10.1186/1471-2164-13-711)
Supplement: Additional file 2 — Table S3. A Synteny table of the pig and human genomes. Table S4. Number of syntenic SNPs of pig and human by each chromosome. Table S5. Number of autosomal pig SNPs mapped onto Sscrofa 10.2 by each chromosome. [file 1471-2164-13-711-S2.pdf]

Table S3. A Synteny table of the pig and human genomes

Pig chromosome	Syntenic human chromosomes
**SSC 1**	HS 6, HS 9, HS 14, HS 15, HS 18
**SSC 2**	HS 5, HS 11, HS 19,
**SSC 3**	HS 2, HS 16, HS 7
**SSC 4**	HS 8, HS 1
**SSC 5**	HS 12, HS 22
**SSC 6**	HS 1, HS 18, HS 16, HS 19
**SSC 7**	HS 14, HS 15, HS 6,
**SSC 8**	HS 4
**SSC 9**	HS 11, HS 7, HS 1
**SSC 10**	HS 10, HS 1
**SSC 11**	HS 13
**SSC 12**	HS 17
**SSC 13**	HS 3, HS 21
**SSC 14**	HS 10, HS 12, HS 1, HS 4, HS 8, HS 22
**SSC 15**	HS 2, HS 8, HS 4
**SSC 16**	HS 5
**SSC 17**	HS 20, HS 4, HS 8
**SSC 18**	HS 7

Table S4. Number of syntenic SNPs of pig and human by each chromosome

**Chr.**	Pig	Human
**1**	1,643	5,740
**2**	1,397	6,581
**3**	1,257	7,375
**4**	1,005	5,262
**5**	1,301	6,340
**6**	1,204	5,480
**7**	730	4,387
**8**	773	3,730
**9**	736	3,699
**10**	878	4,746
**11**	989	4,523
**12**	801	3,937
**13**	417	2,773
**14**	558	2,939
**15**	481	2,133
**16**	370	1,644
**17**	428	1,518
**18**	379	2,223
**19**	103	451
**20**	353	2,043
**21**	157	933
**22**	163	469
**Total**	16,123	78,926

Table S5. Number of autosomal pig SNPs mapped onto Sscrofa 10.2 by each chromosome

CHR1	5,671
CHR2	2,956
CHR3	2,388
CHR4	2,997
CHR5	1,989
CHR6	2,884
CHR7	2,805
CHR8	2,486
CHR9	2,894
CHR10	1,568
CHR11	1,452
CHR12	1,330
CHR13	3,562
CHR14	3,424
CHR15	2,526
CHR16	1,553
CHR17	1,442
CHR18	1,086
Total	45,013
