# Supplementary material for: An integrated approach of comparative genomics and heritability analysis of pig and human on obesity trait: evidence for candidate genes on human chromosome 2
Source: BMC Genomics. 2012 Dec 19;13:711. doi: 10.1186/1471-2164-13-711 (PMC3562524; doi:10.1186/1471-2164-13-711)
Supplement: Additional file 3 — Table S2. Summary of heritability estimates by each chromosome for SUB and BFT traits. [file 1471-2164-13-711-S3.pdf]

Table S2. Summary of heritability estimates by each chromosome for SUB and BFT traits.

Chr.	SUB		BFT	
	h^2	s.e.	h^2	s.e.
**1**	0.009724	0.011464	0.000001	0.024088
**2**	0.031878	0.012597	0.086298	0.038109
**3**	0.000001	0.010411	0.035848	0.026554
**4**	0.015865	0.010786	0.021999	0.023293
**5**	0.008386	0.01033	0.036806	0.029111
**6**	0.010832	0.009881	0.000126	0.015308
**7**	0.003222	0.009119	0.014692	0.021666
**8**	0.005054	0.008771	0.001478	0.017914
**9**	0.00915	0.009095	0.000001	0.01579
**10**	0.016978	0.009875	0.004276	0.016324
**11**	0.025777	0.009858	0.033038	0.028431
**12**	0.013537	0.009668	0.017945	0.022149
**13**	0.009071	0.008478	0.053088	0.029504
**14**	0.006489	0.007624	0.053978	0.032033
**15**	0.011311	0.008212	0.000001	0.020069
**16**	0.004644	0.00784	0.000001	0.014775
**17**	0.002416	0.007088	0.000001	0.013889
**18**	0.005421	0.007659	0.015245	0.019525
**19**	0.000001	0.00606	0.004129	0.010737
**20**	0.000124	0.006529	0.012668	0.017132
**21**	0.000001	0.005413	0.017267	0.019559
**22**	0.007111	0.005531	0.000001	0.007721
