# Supplementary material for: An integrated approach of comparative genomics and heritability analysis of pig and human on obesity trait: evidence for candidate genes on human chromosome 2
Source: BMC Genomics. 2012 Dec 19;13:711. doi: 10.1186/1471-2164-13-711 (PMC3562524; doi:10.1186/1471-2164-13-711)
Supplement: Additional file 4 — Table S6. Major genes predisposing to common obesity identified from multiple previous works and our study. [file 1471-2164-13-711-S4.docx]

Table S6. Major genes predisposing to common obesity identified from multiple previous works and our study.

| Genes | variants a | p-value | Reference |
| --- | --- | --- | --- |
| *FTO* | rs13337356 (16)  H3GA0017791 (6)  INRA0021406 (6) | 0.0157  0.01022  0.007 | [[1-3](#_ENREF_1)] |
| *GNPDA2* | rs7670601 (4)  DIAS0004271 (8) | 0.04617  0.006137 | [[1](#_ENREF_1), [4](#_ENREF_4), [5](#_ENREF_5)] |
| *ADRB3* | MARC0044662 (8) | 0.04111 | [[6-8](#_ENREF_6)] |
| *NPC1* | H3GA0003130 (1) | 0.00002063 | [[2](#_ENREF_2), [9](#_ENREF_9), [10](#_ENREF_10)] |
| *INSIG2* | ASGA0068998 (15) | 0.02239 | [[1](#_ENREF_1), [11](#_ENREF_11), [12](#_ENREF_12)] |

Reference

1. Zhao J, Bradfield JP, Li M, Wang K, Zhang H, Kim CE, Annaiah K, Glessner JT, Thomas K, Garris M: **The Role of Obesity‐associated Loci Identified in Genome‐wide Association Studies in the Determination of Pediatric BMI**. *Obesity* 2012, **17**(12):2254-2257.

2. Meyre D, Delplanque J, Chèvre JC, Lecoeur C, Lobbens S, Gallina S, Durand E, Vatin V, Degraeve F, Proença C: **Genome-wide association study for early-onset and morbid adult obesity identifies three new risk loci in European populations**. *Nat Genet* 2009, **41**(2):157-159.

3. Frayling TM, Timpson NJ, Weedon MN, Zeggini E, Freathy RM, Lindgren CM, Perry JRB, Elliott KS, Lango H, Rayner NW: **A common variant in the FTO gene is associated with body mass index and predisposes to childhood and adult obesity**. *Science* 2007, **316**(5826):889-894.

4. Hotta K, Nakamura M, Nakamura T, Matsuo T, Nakata Y, Kamohara S, Miyatake N, Kotani K, Komatsu R, Itoh N: **Association between obesity and polymorphisms in SEC16B, TMEM18, GNPDA2, BDNF, FAIM2 and MC4R in a Japanese population**. *J Hum Genet* 2009, **54**(12):727-731.

5. Willer CJ, Speliotes EK, Loos RJF, Li S, Lindgren CM, Heid IM, Berndt SI, Elliott AL, Jackson AU, Lamina C: **Six new loci associated with body mass index highlight a neuronal influence on body weight regulation**. *Nat Genet* 2008, **41**(1):25-34.

6. Rankinen T, Zuberi A, Chagnon YC, Weisnagel SJ, Argyropoulos G, Walts B, Pérusse L, Bouchard C: **The human obesity gene map: the 2005 update**. *Obesity* 2012, **14**(4):529-644.

7. Gjesing A, Andersen G, Borch-Johnsen K, Jørgensen T, Hansen T, Pedersen O: **Association of the β< sub> 3</sub>-adrenergic receptor Trp64Arg polymorphism with common metabolic traits: Studies of 7605 middle-aged white people**. *Mol Genet Metab* 2008, **94**(1):90-97.

8. Cieslak J, Nowacka-Woszuk J, Bartz M, Fijak-Nowak H, Grzes M, Szydlowski M, Switonski M: **Association studies on the porcine< i> RETN</i>,< i> UCP1</i>,< i> UCP3</i> and< i> ADRB3</i> genes polymorphism with fatness traits**. *Meat science* 2009, **83**(3):551-554.

9. Walley AJ, Asher JE, Froguel P: **The genetic contribution to non-syndromic human obesity**. *Nature Reviews Genetics* 2009, **10**(7):431-442.

10. Wu L, Xi B, Zhang M, Shen Y, Zhao X, Cheng H, Hou D, Sun D, Ott J, Wang X: **Associations of six single nucleotide polymorphisms in obesity-related genes with BMI and risk of obesity in Chinese children**. *Diabetes* 2010, **59**(12):3085-3089.

11. Herbert A, Gerry NP, McQueen MB, Heid IM, Pfeufer A, Illig T, Wichmann HE, Meitinger T, Hunter D, Hu FB: **A common genetic variant is associated with adult and childhood obesity**. *Science* 2006, **312**(5771):279-283.

12. Mutch DM, Clément K: **Unraveling the genetics of human obesity**. *PLoS genetics* 2006, **2**(12):e188.
