# Supplementary material for: An integrated approach of comparative genomics and heritability analysis of pig and human on obesity trait: evidence for candidate genes on human chromosome 2
Source: BMC Genomics. 2012 Dec 19;13:711. doi: 10.1186/1471-2164-13-711 (PMC3562524; doi:10.1186/1471-2164-13-711)

Figure S1. Graphical summary (Manhattan plot) of genome-wide association results of SUB (above) and BFT. RASAL2, known as obesity gene, was significantly associated in SUB trait of pig.

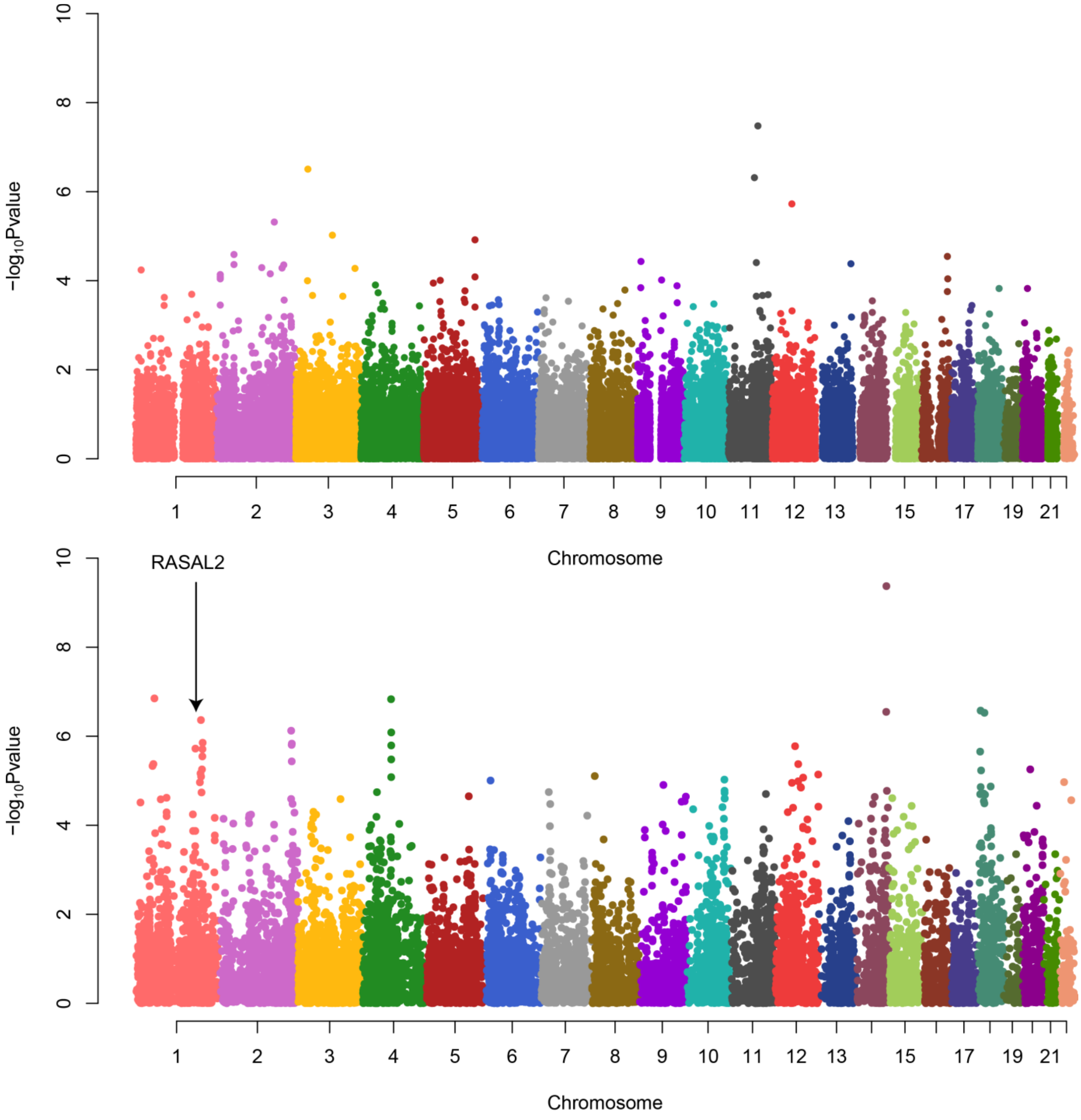

Supplement: Additional file 5 — Figure S1. Graphical summary (Manhattan plot) of genome-wide association results of SUB (above) and BFT. RASAL2, known as obesity gene, was significantly associated in SUB trait of pig. [file 1471-2164-13-711-S5.pdf]
